# Silicalite-1/PDMS Hybrid Membranes on Porous PVDF Supports: Preparation, Structure and Pervaporation Separation of Dichlorobenzene Isomers

**DOI:** 10.3390/polym14091680

**Published:** 2022-04-21

**Authors:** Qiuping He, Wei Chen, Pengfei Wang, Xiaoming Dou

**Affiliations:** 1 Institute of Photonics & Bio-Medicine, School of Science, East China University of Science and Technology, 130 Meilong Road, Shanghai 200237, China; tech@sh-lq.com; 2 Shanghai Lvqiang New Materials Co., Ltd., 258 Hengle Road, Shanghai 201806, China; 3 State Key Laboratory of Polyolefin Catalytic Technology and High Performance Material, Shanghai Research Institute of Chemical Industry Co., Ltd., 345 Yunling East Road, Shanghai 200062, China

**Keywords:** silicalite-1 zeolites, PDMS, hybrid membranes, pervaporation, dichlorobenzene isomer separation

## Abstract

Separation of dichlorobenzene (DCB) isomers with high purity by time− and energy−saving methods from their mixtures is still a great challenge in the fine chemical industry. Herein, silicalite-1 zeolites/polydimethylsiloxane (PDMS) hybrid membranes (silicalite-1/PDMS) have been successfully fabricated on the porous polyvinylidene fluoride (PVDF) supports to first investigate the pervaporation separation properties of DCB isomers. The morphology and structure of the silicalite-1 zeolites and the silicalite-1/PDMS/PVDF hybrid membranes were characterized by XRD, FTIR, SEM and BET. The results showed that the active silicalite-1/PDMS layers were dense and continuous without any longitudinal cracks and other defects with the silicalite-1 zeolites content no more than 10%. When the silicalite-1 zeolites content exceeded 10%, the surfaces of the active silicalite-1/PDMS layers became rougher, and silicalite-1 zeolites aggregated to form pile pores. The pervaporation experiments both in single-isomer and binary−isomer systems for the separation of DCB isomers was further carried out at 60 °C. The results showed that the silicalite-1/PDMS/PVDF hybrid membranes with 10% silicalite-1 zeolites content had better DCB selective separation performance than the silicalite-1/α−Al_2_O_3_ membranes prepared by template method. The permeate fluxes of the DCB isomers increased in the order of m−DCB < o−DCB < p−DCB both in single-isomer and binary-isomers solutions for the silicalite-1/PDMS/PVDF hybrid membranes. The separation factor of the silicalite-1/PDMS/PVDF hybrid membranes for p/o−DCB was 2.9 and for p/m−DCB was 4.6 in binary system. The permeate fluxes of the silicalite-1/PDMS/PVDF hybrid membranes for p−DCB in p/o−DCB and p/m−DCB binary−isomers solutions were 126.2 g∙m^−2^∙h^−1^ and 104.3 g∙m^−2^∙h^−1^, respectively. The thickness−normalized pervaporation separation index in p/o−DCB binary−isomers solutions was 4.20 μm∙kg∙m^−2^∙h^−1^ and in p/m−DCB binary−isomers solutions was 6.57 μm∙kg∙m^−2^∙h^−1^. The results demonstrated that the silicalite-1/PDMS/PVDF hybrid membranes had great potential for pervaporation separation of DCB from their mixtures.

## 1. Introduction

Dichlorobenzene (DCB) isomers including p−dichlorobenzene (p−DCB), o−dichlorobenzene (o−DCB) and m-dichlorobenzene (m−DCB), are extensively used as important intermediates in the production of pesticides, medicines, fragrances, pigments and dyes, especially p−DCB and o−DCB [1,2,3,4,5,6]. Generally, the DCB isomers are prepared by directional catalytic chlorination of benzene or mono−chlorobenzene in both vapor and solvent system [7,8]. However, the above two methods always produce a mixture of the three DCB isomers. Compared with DCB isomer mixtures, the individual DCB has a higher added value. Therefore, it is quite important to separate the individual ingredient from the DCB isomer mixtures. Industrially, the combination of extract distillation and crystallization methods have been successfully used for recovery of the DCB isomers [9,10,11,12]. Nevertheless, the close boiling points and similar molecule structures of these isomers make the traditional separation method, a time− and energy−consuming process. Owing to the regular crystal structure, excellent pore system and special surface properties, zeolites have been particularly used in separation, purification and catalysis [13,14,15,16,17,18,19]. Recently, the MFI−type zeolites adsorption technology (also granular silicalite-1 zeolites adsorbents) for the separation of DCB isomers has received more attention because of its simple operation and high efficiency [5,19,20,21]. However, the disadvantages of large mass transfer resistance, low zeolite utilization, high thermal effect and difficulty in desorption limit its future industrial application in DCB isomers separation field. Moreover, the appearance of non-adsorbed pores during zeolites molding reduces the selective separation performance of DCB isomers, which were unable to meet the higher purity requirements of the individual DCB for high−end DCB derivatives. Therefore, the development of an economical and efficient technology for separation of high−purity DCB is quite important. Due to the low mass transfer resistance, low energy consumption, high zeolites utilization and efficiency, zeolite membranes (inorganic and polymer) have been widely used in many fields such as chemical, medicine, environmental and other industrial fields [21,22,23,24,25,26,27,28]. The uniform narrow molecular−sized pore distribution makes them attractive as new shape−selective materials for gas and liquid separation [29,30,31,32], especially for isomeric and close−boiling mixtures [33,34,35], such as DCB isomers separation. Fortunately, in our previous study, we have successfully adopted dense and continuous silicalite-1/α−Al_2_O_3_ membranes for the pervaporation separation of DCB from binary−isomers solutions [3]. Despite the template−free method, fabricated silicalite-1/α−Al_2_O_3_ membranes exhibit excellent DCB pervaporation separation properties, the large−scale preparation of the silicalite-1/α−Al_2_O_3_ with structural integrity is still a huge technical difficulty [36,37], which seriously hinders its future industrial application in DCB separation field. To our knowledge, the preparation of dense and continuous zeolites–polymer hybrid membranes is simpler and more controllable than inorganic zeolites membranes, which providing a new possible strategy for DCB separation. To date, many zeolites–polymer hybrid membranes such as MFI−type zeolites/polydimethylsiloxane (PDMS) hybrid membranes [38,39,40,41], CHA−type zeolites/polytrimethylsilyl−1−propyne (PTMSP) hybrid membranes [42,43], FAU−type zeolites/poly (ether block amide) (PEBA) hybrid membranes [44,45,46,47], and LTA−type zeolites/polyvinyl alcohol (PVA) hybrid membranes have been successfully prepared [48,49,50]. Among the above zeolites–polymer hybrid membranes, MFI-type zeolites/PDMS hybrid membranes are widely used in gas and liquid separation because of the stable structure, excellent corrosion resistance and easy film−forming properties [38,51,52,53,54,55]. Wang et al. utilized nanosized silicalite-1 zeolites with the modification by silane coupling agents to prepare the silicalite-1/PDMS hybrid membranes for enhancing ethanol/water pervaporation separation performance [56]. Xue et al. adopted ZSM−5 zeolites with different Si/Al ratios to prepare ZSM−5/PDMS hybrid membranes for pervaporation recovery of butanol [57]. Pattabhi Ramaiah prepared ZSM−5 zeolites loaded PDMS hybrid membranes consisting of three hydrophobic layers for the removal of hazardous chlorinated VOCs from aqueous solutions [58]. Wu et al. synthesized ZSM−5/PDMS hybrid membranes for pervaporation separation of acetaldehyde from aqueous solutions [59]. Banihashemi et al. prepared ZSM−5/PDMS hybrid membranes by nanosized silicalite-1 zeolites powders for separation of CO_2_ from CO_2_/CH_4_ or CO_2_/N_2_ system [60]. Despite the above research, there is still no report on the pervaporation separation of DCB isomers by using silicalite-1/PDMS hybrid membranes. Herein, in the present work, we have successfully prepared dense and continuous silicalite-1/PDMS hybrid membranes on the porous PVDF supports (denote as silicalite-1/PDMS/PVDF) for, firstly, pervaporation separation of DCB isomers. To achieve this, the silicalite-1/PDMS hybrid membranes were prepared by the following steps: (i) preparation of high viscosity silicalite-1−filled PDMS solutions by mixing hydroxyl−terminated PDMS, n−heptane, silicalite-1 powders, TEOS and DBTOL; (ii) casting the silicalite-1−filled PDMS solutions on the surfaces of the porous PVDF supports and scraping to obtain wet silicalite-1/PDMS hybrid membranes; (iii) cross−linked under vacuum as well as heating to obtain silicalite-1/PDMS/PVDF hybrid membranes. The main objectives of this study are to fabricate silicalite-1/PDMS/PVDF hybrid membranes with dense and continuous silicalite-1/PDMS layers without any cracks and flaws as well as to study the morphology, structure and selective separation performance of DCB isomers. The morphology and structure of silicalite-1/PDMS/PVDF hybrid membranes were characterized by XRD, FTIR, SEM and BET. The pervaporation measurements both in single−isomer and binary−isomer systems for the separation of DCB were further carried out. For comparison, the dense and continuous silicalite-1/α−Al_2_O_3_ membranes prepared by template method for pervaporation separation of DCB isomers were also investigated. The above studies would provide fundamental technical and data support for the application of silicalite-1/PDMS/PVDF hybrid membranes in separation of pure DCB from DCB isomers mixture in future industry.

## 2. Materials and Methods

### 2.1. Materials and Reagents

The hydroxyl−terminated polydimethylsiloxane (OH-PDMS) with kinetic viscosity of 20,000 MPa∙s was purchased from Beijing Dingye Co., Ltd. (Beijing, China). Tetraethylorthosilicate (TEOS, 98%, AR), n−heptane (99%, AR), p−dichlorobenzene (99%, AR), o−dichlorobenzene (98%, AR), m−dichlorobenzene (99%, AR) and anhydrous ethanol (99.5%, AR) were all purchased from Aladdin Chemical Reagents Co., Ltd. (Shanghai, China). Dibutyltin dilaurate (DBTOL, 95%, AR) was purchased from Sinopharm Chemical Reagents Co., Ltd. (Shanghai, China). Silicalite-1 zeolites powders were purchased from Nankai University catalyst Co., Ltd. (Tianjing, China). Polyvinylidene fluoride (PVDF) supports (hydrophobic, diameter 80 mm, thickness 0.15 mm) with an average pore size of 0.22 μm were purchased from Hangzhou Micropai Technology Co., Ltd. (Hangzhou, China). All the chemicals were used without any further purification.

### 2.2. Preparation of Silicalite-1/PDMS/PVDF Hybrid Membranes

Calcination of silicalite-1 zeolites: In order to void the residual templates and adsorbed moisture, the silicalite-1 zeolites should be calcined in atmosphere furnace with a heating rate of 3 °C/min, and held at 550 °C for 2.0 h, and then slowly cooled down to room temperature for further utilization. Hybrid membranes preparation: The solutions with the compositions of 10 OH−PDMS: (75–95) n−heptane: 1TEOS: 0.2 DBTOL for scraping of silicalite-1/PDMS hybrid membranes were prepared by successively mixing OH−PDMS pre−polymer, n−heptane, TEOS and DBTOL according to the following process [61,62]. Firstly, 10 g of OH−PDMS pre−polymer and 75 g n−heptane were well mixed under vigorous stirring for 4 h. Secondly, 1 g calcined silicalite-1 zeolites powders were added into the above solutions for another 1 h stirring, and then sonicated for 0.5 h to achieve uniform dispersion of silicallite−1 zeolites in PDMS solutions. After the treatment, 1 g cross−linker TEOS were added and agitated for 1 h. Lastly, 0.2 g catalyst DBTOL were added to the mixture and vigorous stirring for 1–3 h. As the suspension became highly viscous without bubbles, it was immediately cast on the porous PVDF supports and subsequently scraped to form wet and thin silicalite-1/PDMS hybrid membranes. The wet hybrid membranes were kept at room temperature for 12–24 h to evaporate the extra solvents and placed in a vacuum oven at 80 °C for 6 h to assure complete crosslink, then the dense and continuous silicalite-1/PDMS/PVDF hybrid membranes with 10% zeolites content (based on the PDMS weight) were obtained. To prepare silicalite-1/PDMS/PVDF hybrid membranes with 5–30% zeolites contents, we further changed the amount of n−heptane and silicalite-1 zeolites from 55–85 g and 0.5–3 g, respectively.

### 2.3. Characterization

The morphologies of silicalite-1 zeolites, PDMS/PVDF membranes and silicalite-1/PDMS/PVDF hybrid membranes were determined with scanning electron microscopy (SEM, Hitachi S−4800, CamScan, Tokyo, Japan) equipped with W−Tungsten filament, operated at 3 kV. The fractured section of PDMS/PVDF membranes and silicalite-1/PDMS/PVDF hybrid membranes were fractured in liquid nitrogen. The crystalline phases of silicalite-1 zeolites, PDMS/PVDF membranes and silicalite-1/PDMS/PVDF hybrid membranes were examined with X−ray powder diffraction (XRD, D/max-II B, Tokyo, Japan) using CuKα radiation (λ = 1.541874 Å) within the scanning range of 3° to 80° at a scanning rate of 6° min^−1^ and a step size of 0.02°. The porosities of silicalite-1 zeolites were determined by a Micrometrics ASAP 2020 nitrogen adsorption analyzer. The samples were degassed at 350 °C for 4 h under vacuum overnight prior to the measurement at −196 °C. The micropore and mesopore size distribution of the samples were estimated by Density Functional Theory (DFT) and Barrett–Joyner–Halenda (BJH) methods, respectively. The external surface area and the micropore volume were determined using t-plot technique. The FTIR spectrums of silicalite-1 zeolites, PDMS/PVDF membranes and silicalite-1/PDMS/PVDF hybrid membranes were recorded on a Nicolet Impact 410 FTIR spectrometer equipped with a DTGS detector by using the KBr pellet technique in the range 400~4000 cm^−1^.

### 2.4. Pervaporation Evaluation Experiment

**Scheme 1:**
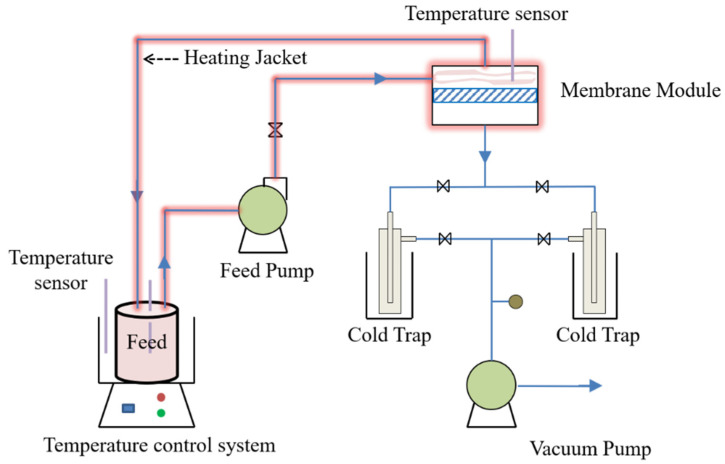
Illustration of the pervaporation experiment process.

The pervaporation experiment including feed circulation, heating, condensation and vacuum system shown in Scheme 1, which were conducted with a self−designed apparatus described in our previously study [3]. The membrane module provided an effective diameter of 50 mm and area of 19.63 cm^2^. The permeate side pressure was adjusted to 1000 Pa by a precision vacuum pump. The silicalite-1/PDMS/PVDF hybrid membranes with a diameter of 50 mm circle were installed in the membrane module, and then sealed with O-rings for measurement. Before evaluation, the DCB solutions was heated and maintained at 60 °C with water bath. Subsequently, the single−isomer or binary−isomers solutions (of equal molar content) was continuously circulated from a feed tank to the membrane module via the peristaltic pump, and the flow rate was maintained at 100 mL/min. On the permeate side, the DCB permeation was collected in the cold traps and condensed by liquid nitrogen (−196 °C). At the given time intervals, the condensed DCB permeation liquids were analyzed by GC with an online flame ionization detector (FID). Membranes separation performance was evaluated on the basis of the total flux (*J*), the selective separation factor (α), the thickness−normalized pervaporation flux (*J_N_*) and the thickness−normalized pervaporation separation index (*PSI_N_*).

The total permeate flux (*J*) was calculated according to the following equations:

$$J=\frac{W}{A\times t}$$

where *W* (g) is the weight of the collected DCB isomers permeation, *A* (m^2^) is the effective area of the silicalite-1/PDMS/PVDF hybrid membranes, and *t* (h) is the permeation time.

The selective separation factor (α_p/o_ or α_p/m_) in binary system was calculated according to the following equations:

$$\alpha_{p/o}=\frac{y_{p-\mathrm{DCB}/}y_{o-\mathrm{DCB}}}{x_{p-\mathrm{DCB}/}x_{o-\mathrm{DCB}}}$$

$$\alpha_{p/m}=\frac{y_{p-\mathrm{DCB}/}y_{m-\mathrm{DCB}}}{x_{p-\mathrm{DCB}/}x_{m-\mathrm{DCB}}}$$

where *y*_p−DCB_, *y*_o−DCB_ and *y*_m−DCB_ represent the initial weight fraction of p−DCB, o−DCB and m−DCB in feed, respectively; *x*_p−DCB_, *x*_o−DCB_ and *x*_m−DCB_ represent the weight fraction of p−DCB, o−DCB and m−DCB in permeate solution, respectively. All experiment data are the average of duplicate determinations, and the relative error is below 3.0%.

The thickness-normalized total pervaporation flux (*J_N_*_,*p/o−DCB*_), the thickness−normalized pervaporation flux of p−DCB (*J_N_*_,*p−DCB*_), the thickness−normalized pervaporation flux of o−DCB (*J_N_*_,*o−DCB*_), and the thickness−normalized pervaporation separation index (*PSI_N,p/o−DCB_*) in p/o−DCB binary−isomers solutions were calculated according to the following equations:

$$J_{N,p/o-DCB}=J_{t,p/o-DCB}\times d$$

$$J_{N,p-DCB}=J_{p,p/o-DCB}\times d_{\;}$$

$$J_{N,o-DCB}=J_{o,p/o-DCB}\times d_{\;}$$

$$PSI_{N,p/o-DCB}=J_{N,p-DCB}\times {\left(\alpha -1\right)}_{\;}$$

The thickness−normalized total pervaporation flux (*J_N_*_,*p/m−DCB*_), the thickness−normalized pervaporation flux of p−DCB (*J_N_*_,*p−DCB*_), the thickness−normalized pervaporation flux of m−DCB (*J_N_*_,_*_m−DCB_*), and the thickness−normalized pervaporation separation index (*PSI_N_*) in p/m−DCB binary−isomer solutions were calculated according to the following equations:

$$J_{N,p/m-DCB}=J_{t,p/m-DCB}\times d$$

$$J_{N,p-DCB}=J_{p,p/m-DCB}\times d$$

$$J_{N,m-DCB}=J_{m,p/m-DCB}\times d$$

$$PSI_{N,p/m-DCB}=J_{N,p-DCB}\times \left(\alpha -1\right)$$

where *d* (μm) was the thickness of the membranes; *J_t_*_,*p*/*o−DCB*_ (kg∙m^−2^∙h^−1^), *J_p_*_,*p*/*o−DCB*_ (kg∙m^−2^∙h^−1^) and *J_o_*_,*p*/*o−DCB*_ (kg∙m^−2^∙h^−1^) were the total permeate flux, p−DCB permeate flux and o−DCB permeate flux in p/o−DCB binary−isomer solutions, respectively. *J_t_*_,*p*/*m−DCB*_ (kg∙m^−2^∙h^−1^), *J_p_*_,*p*/*m−DCB*_ (kg∙m^−2^∙h^−1^) and *J_m_*_,*p*/*m−DCB*_ (kg∙m^−2^∙h^−1^) were the total permeate flux, p−DCB permeate flux and m−DCB permeate flux in p/m−DCB binary−isomer solutions, respectively.

## 3. Results

### 3.1. Morphology and Structure of Silicalite-1/PDMS/PVDF Hybrid Membranes

**Figure 1:**
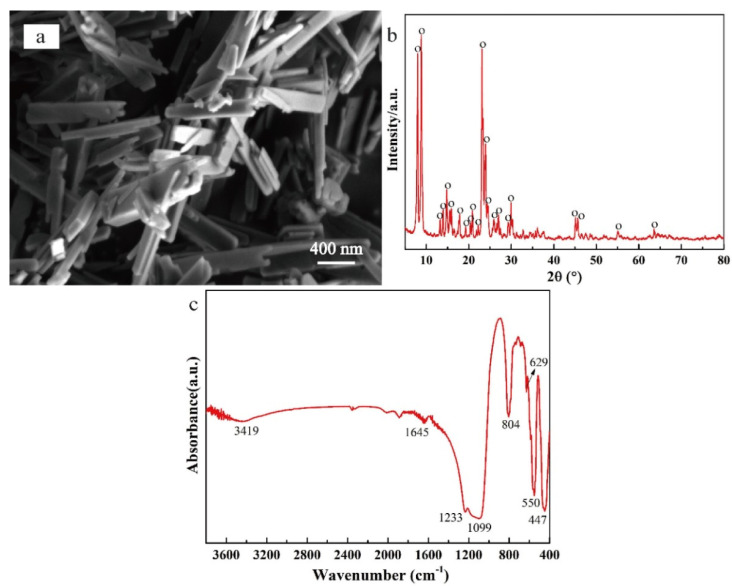
SEM images (**a**), XRD patterns (**b**) and FTIR spectrums (**c**) of silicalite-1 zeolites powders.

As we known, the molecular size of p−DCB (~0.58 nm), o−DCB (~0.68 nm) and m−DCB (~0.68 nm) are close to the pore size of MFI−type zeolites [6]. Hence, the MFI−type zeolites are often used as adsorption material to separate the DCB isomers. Theoretically, the pore size of the MFI−type zeolites becomes smaller with the framework Si/Al ration increasing [5]. Among the various MFI−type zeolites, silicalite-1 zeolites have more uniform and narrower pore size distribution, which is more suitable for DCB isomers separation. Fortunately, the industrialization of silicalite-1 zeolites has been successfully realized in recent years, which was beneficial for the fabrication of zeolites–polymer hybrid membranes. The morphology and structure of the silicalite-1 zeolite powders were checked by SEM images, XRD patterns and FTIR spectrums as shown in Figure 1. The SEM images given in Figure 1a revealed the morphology of silicalite-1 zeolites particles. It was noted that the silicalite-1 zeolites exhibited rod−like morphology with a length below 1.5 μm, width below 200 nm and thickness below 60 nm, respectively. The integrity of single crystal structure indicated that the silicalite-1 zeolites had high crystallinity. Figure 1b,c depicted the XRD patterns and FTIR spectrums of the silicalite-1 zeolite powders. As shown in Figure 1b, only the characteristic peaks of the MFI−type zeolites were detected in silicalite-1 zeolite powders, such as typical diffraction plane at 2θ = 7.896°, 8.810°, 13.272°, 13.889°, 14.691°, 23.025°, 23.333° and 24.012°. The high intensity of the peaks indicated that the silicalite-1 zeolites were well crystallized, and the relative crystallinity was about 99% according to the typical diffractions (based on silicalite-1 zeolites−EKZ). For the FTIR spectrums of the silicalite-1 zeolites particles illustrated in Figure 1c, the broad bands between 3200~3600 cm^−1^ were the O−H bond stretching vibration, and the broad bands between 1600~1700 cm^−1^ were the O−H bond bending vibration of the absorbed H_2_O [39,63]. The bands at 550 cm^−1^ and 1233 cm^−1^ corresponded to the five−membered ring chains and different ring structure [64]. The sharp bands at 447 cm^−1^, 804 cm^−1^ and 1099 cm^−1^ were ascribed to the bending vibration, symmetric stretching and asymmetric stretching of Si−O−Si, respectively [65]. The XRF result further demonstrated that there was no any Al existence in the silicalite-1 zeolites powders (data not shown).

**Figure 2:**
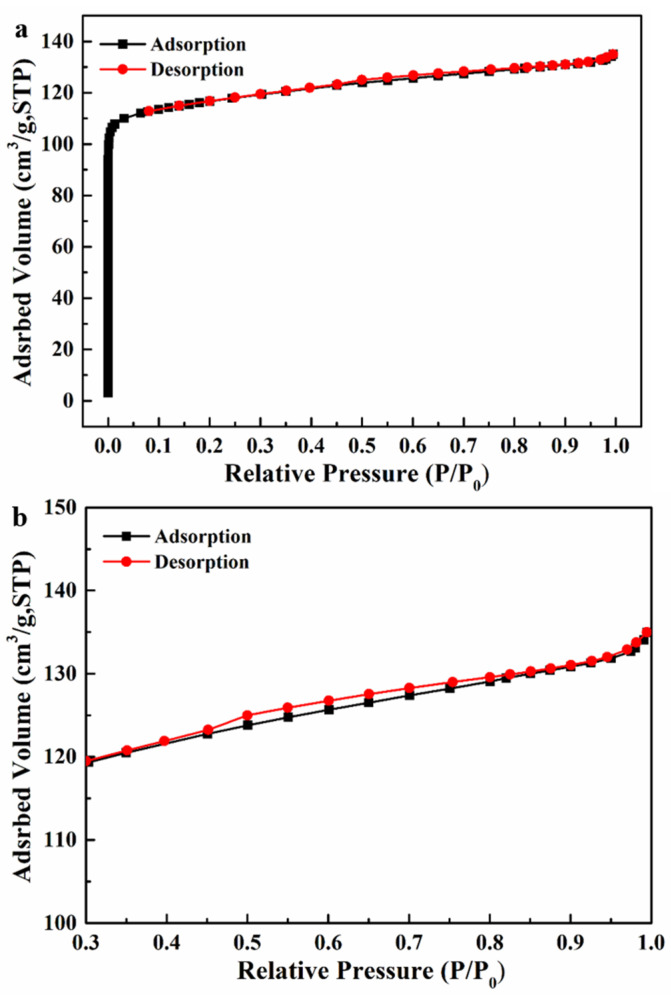
Nitrogen adsorption and desorption isotherm curves of silicalite-1 zeolites powders. (**b**) was the magnified segment of (**a**).

The porosities of the silicalite-1 zeolites powders were further studied by N_2_ sorption measurements, as shown in Figure 2. Notably, a rapidly rise curve was exhibited at low partial pressure (P/P_0_ = 0–0.1, Figure 2a). It proved that the centralized distribution of microporous were existed in the silicalite-1 zeolites powders. The N_2_ adsorption/desorption isotherm for the silicalite-1 zeolites powders was quite close to the typical type−I isotherm [66,67]. It further indicated that abundant microporous were existed in the silicalite-1 zeolites powders. Interestingly, an extremely weak hysteresis loop (Figure 2b) was detected at high partial pressure (P/P_0_ = 0.45–0.85), which could be associated with the presence of inter−particle mesoporous [68,69]. The results from the N_2_ sorption measurements were summarized in Table 1. The BET specific surface and micropore area the silicalite-1 zeolites based on the N_2_ sorption measurements were 356.1 m^2^/g and 296.4 m^2^/g, respectively. Moreover, the total pore volume and micropore volume of the silicalite-1 zeolites were 0.209 cm^3^/g and 0.171 cm^3^/g, respectively. The higher area and volume of the micropore than that of mesoporous indicated that the silicalite-1 zeolites might has better separation property than other MFI−type zeolites.

**Figure 3:**
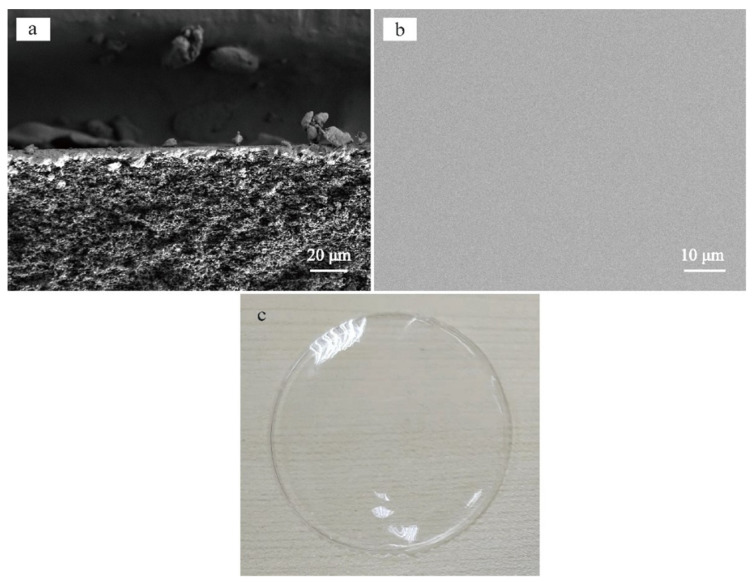
SEM images of the PDMS/PVDF membranes and photographs of the pure PDMS membranes without coating on PVDF supports: (**a**) cross-sectional SEM images; (**b**) top-view SEM images; and (**c**) photographs of the PDMS membranes.

Figure 3 were the SEM images and photographs of the PDMS/PVDF membranes. The thickness of the active PDMS layers on the surfaces of the porous PVDF supports was about 3~5 μm without any longitudinal cracks (Figure 3a). The further top−view SEM images demonstrated that the surfaces of PDMS layers were smooth and dense without any appreciable pores and other flaws (Figure 3b). The dense and continuous PDMS layers without any defects ensured no liquid leakage in following separation of DCB isomers by silicalite-1/PDMS/PVDF hybrid membranes. The photographs of the pure PDMS membranes without coating on the porous PVDF supports were illustrated in Figure 3c. The PDMS membranes were transparent with poor mechanical properties.

**Figure 4:**
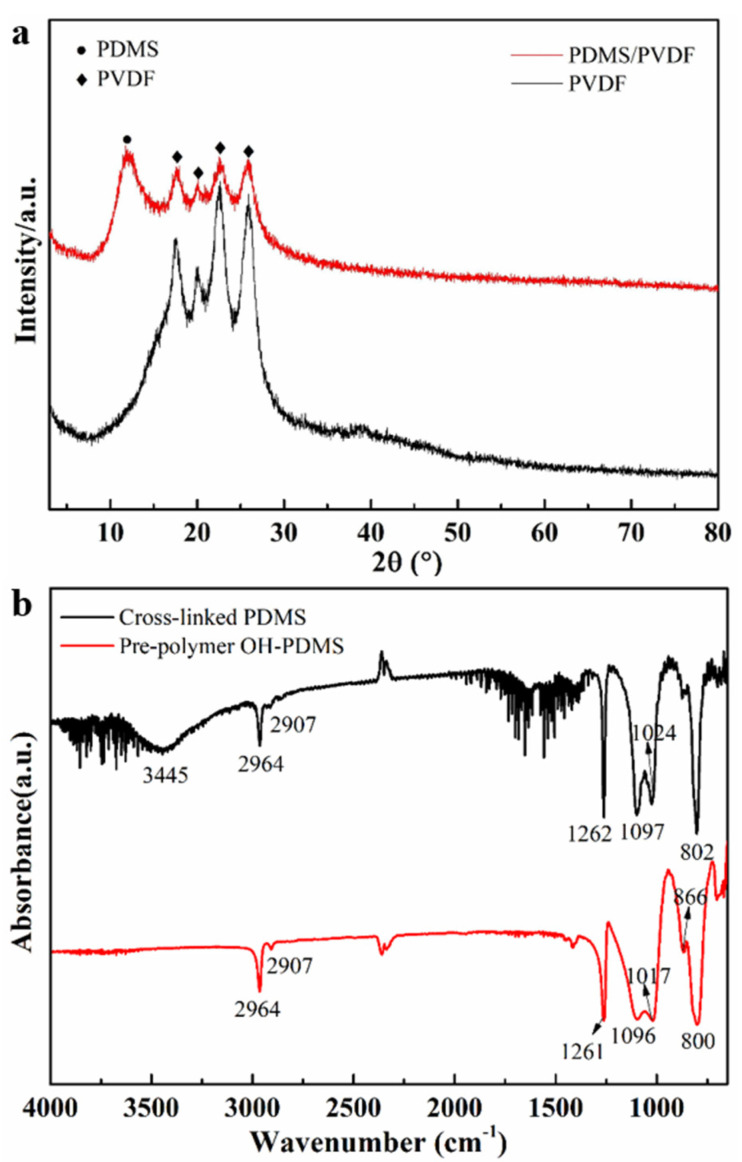
XRD patterns and FTIR spectrums of the PDMS membranes: XRD patterns (**a**); FTIR spectrums (**b**).

The XRD patterns of the PDMS/PVDF membranes were exhibited in Figure 4a. It was noted that a broad peak corresponding to the diffraction of the PDMS at 2θ from 10 to 15° was detected, which was similar to the peak reported in the previous literature [70]. This characteristic peak indicated that the pre−polymer OH−PDMS successfully cross−linked with TEOS. Moreover, the characteristic peaks attributed to the porous PVDF supports were detected at 2θ from 18° to 30°. The varieties of functional groups before and after PDMS cross−linking were further characterized by the FTIR spectrums (Figure 4b). As illustrated in Figure 4b, there was no any new peak detected before and after crosslinking. The bands at 2964 cm^−1^ and 2907 cm^−1^ represented the asymmetrical stretching vibration and stretching vibration of CH_3_ [71,72] and were both detected in the FTIR spectrums of the cross-linked PDMS and uncross−linked OH−PDMS, respectively. For the pre−polymer OH-PDMS, the band appearing at 1261 cm^−1^ was the stretching vibration of Si−CH_3_, and the band at 801 cm^−1^ was the stretching vibration of Si−CH_3_ and rocking vibration of CH_3_ [72,73]. The peak located at the region near 1017 cm^−1^ was the asymmetrical stretching vibration of Si−O−Si, while the band at 1096 cm^−1^ was ascribed to the stretching vibration of Si−OH [72,74]. Moreover, the weak band at 866 cm^−1^ ascribed to the stretching vibration of Si−CH_3_ was also detected [75]. For the cross−linked PDMS, the broad band around 3445 cm^−1^ was the O−H stretching vibration of the adsorbed H_2_O [58]. Similar to the FTIR spectrums of the pre−polymer OH−PDMS, the band appearing at 1262 cm^−1^ attributed to the stretching vibration of Si−CH_3_, and the band at 802 cm^−1^ ascribed to the combination of Si−CH_3_ stretching vibration and CH_3_ rocking vibration [72,73]. Notably, the characteristic peaks near the region of 1262 cm^−1^, 1000–1100 cm^−1^ and 802 cm^−1^ became sharper after crosslinking. Meanwhile, the peaks at 866 cm^−1^ became weaker. These varieties indicated that the O−H bond interacted with the –O−C_2_H_5_ bond of the cross−linker TEOS to form Si−O−Si under the catalyzing of DBTOL [76]. Hence, both the bands at 1024 cm^−1^ and 1097 cm^−1^ were the asymmetrical stretching vibration of Si−O−Si. Moreover, after crosslinking, there was a 7 cm^−1^ upward shift of the asymmetrical stretching vibration bond at 1024 cm^−1^, indicating that additional interaction between the SiO_4_ derived from TEOS and the Si−O−Si in PDMS. The FTIR spectrum further demonstrated that the pre−polymer OH−PDMS has been well cross−linked, which was in accordance with the results of the SEM images and XRD patterns.

**Figure 5:**
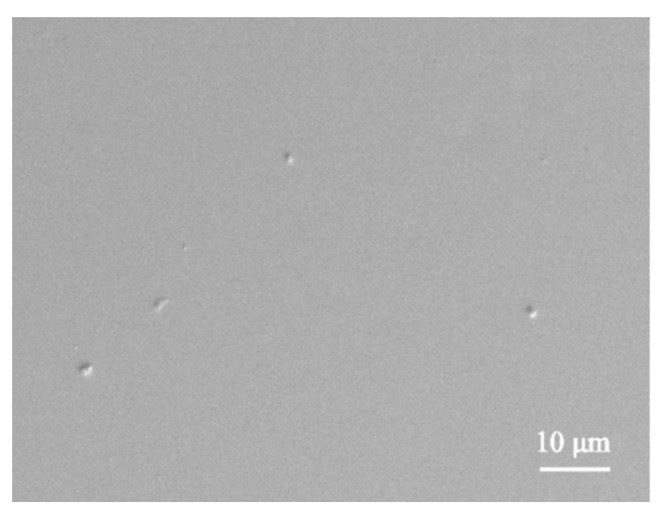
The top-view SEM image of the PDMS/PVDF membranes after treatment with DCB isomers (molar ration 1:1) at 60 °C for 5 days.

As an ideal material for the separation of DCB isomers, the materials must have good compatibility with the solutions. Herein, we used the mixture solutions of p−DCB and o−DCB (molar ration 1:1) to verify the stability of the PDMS membranes. The compatibility experiment was carried out by a static purification test as the following operation. The PDMS/PVDF membranes were immersed in the above mixture solutions with the temperature of 60 °C for 5 days, the top−view SEM image of the DCB isomers treated PDMS/PVDF membranes was shown in Figure 5. Obviously, the PDMS/PVDF membranes after treated with DCB isomers solutions also exhibited similar smooth and dense surfaces without any corrosion traces (compared with Figure 3b). This result indicated that the PDMS had a better stability contacting with the DCB isomers.

**Figure 6:**
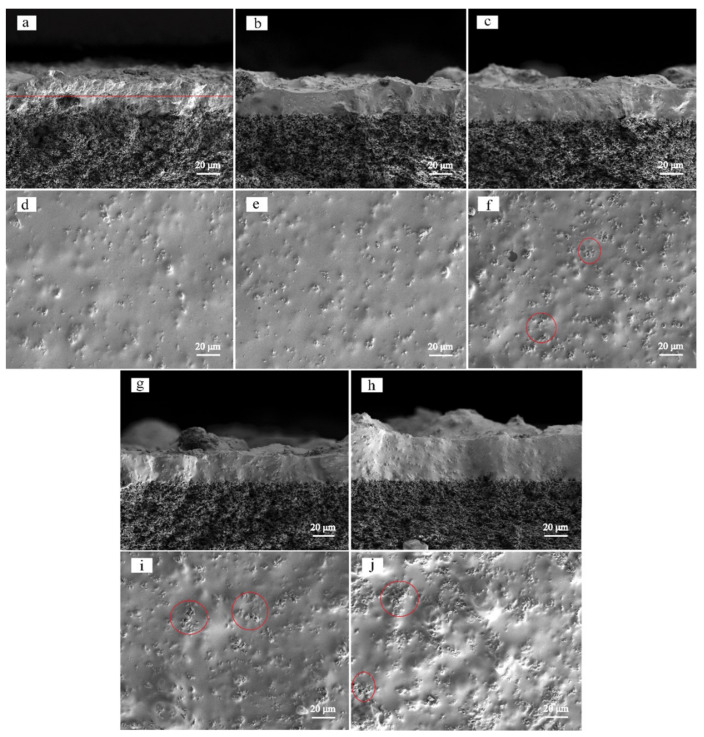
SEM images of the silicalite-1/PDMS/PVDF hybrid membranes with different zeolites contents: (**a**,**d**) 5% loading; (**b**,**e**) 10% loading; (**c**,**f**) 15% loading; (**g**,**i**) 20% loading; and (**h**,**j**) 30% loading. (**a**–**c**,**g**,**h**) Cross-sectional SEM images; and (**d**–**f**,**I**,**j**) top-view SEM images.

Silicalite-1/PDMS/PVDF hybrid membranes might have great potential for separation of the individual DCB from DCB isomers mixtures because they exhibit the following advantages. Firstly, the flake membranes structure significantly reduces the mass transfer resistance, increases the contact areas between the DCB solutions and silicalite-1 zeolites; secondly, the dense and continuous silicalite-1/PDMS layers on the porous PVDF supports without any non−adsorption defects and the centralized micropore distribution of silicalite-1 zeolites ensure the separation of DCB; thirdly, if the silicalite-1/PDMS/PVDF hybrid membranes serve as replacement for the traditional granular silicalite-1 zeolites adsorbent, the simple operation process and low regeneration energy consumption will be achieved. In order to prepare the silicalite-1/PDMS/PVDF hybrid membranes, the calcined silicalite-1 zeolites powders was uniformly dispersed in high viscosity PDMS solutions, and then scraped to form a dense and continuous silicalite-1/PDMS layers on the surfaces of the porous PVDF supports after sufficient cross−linking. The morphology and structure of the silicalite-1/PDMS/PVDF hybrid membranes were further investigated by SEM images, XRD patterns and FTIR spectrums. Figure 6 revealed the cross−sectional and top−view images of the silicalite-1/PDMS/PVDF hybrid membranes with different zeolites contents. As mentioned in Figure 6a–c,g,h, it appeared that the active silicalite-1/PDMS layers were tightly and properly adhered on the surfaces of the porous PVDF supports. When the silicalite-1 zeolites loading was 5%, the thickness of the active silicalite-1/PDMS layers was about 10–15 μm (Figure 6a), and no longitudinal cracks were detected. As shown in Figure 6d, the silicalite-1 zeolites homogeneously distributed in the active silicalite-1/PDMS layers, and the surfaces of the active silicalite-1/PDMS layers was flat and dense without any pinholes. When the silicalite-1 zeolites loading increased to 10%, there were also no any longitudinal cracks (Figure 6b) and surfaces pinholes (Figure 6e) existed in the active silicalite-1/PDMS layers. The thickness of the active silicalite-1/PDMS layers with 10% silicalite-1 zeolites loading was about 15–20 μm (Figure 6b). When the silicalite-1 zeolites loading increased to 15%, still no longitudinal cracks were detected (Figue 6c), and the thickness of the active silicalite-1/PDMS layers reached 20–25 μm. Notably, compared with 5% and 10% silicalite-1 zeolites loading, the surfaces of the active silicalite-1/PDMS layers became rougher and some silicalite-1 zeolites particles piled up together to form pores (red circle). The formation of these pile pores was not conducive to the separation of DCB isomers. However, as the silicalite-1 zeolites loading further increase, the thickness of the silicalite-1/PDMS layers became thicker and the silicalite-1 zeolites particles were obviously aggregation (Figure 6i,j). The aggregation of the silicalite-1 zeolites formed more pile pores. The thickness of silicalite-1/PDMS layers with 20% and 30% silicalite-1 zeolites loading were about 25–30 μm and 35–40 μm, respectively. Conceivably, the dense and continuous silicalite-1 zeolites layers without any defects and zeolites aggregation on silicalite-1/PDMS/PVDF hybrid membranes might have excellent separation performance of DCB isomers.

**Figure 7:**
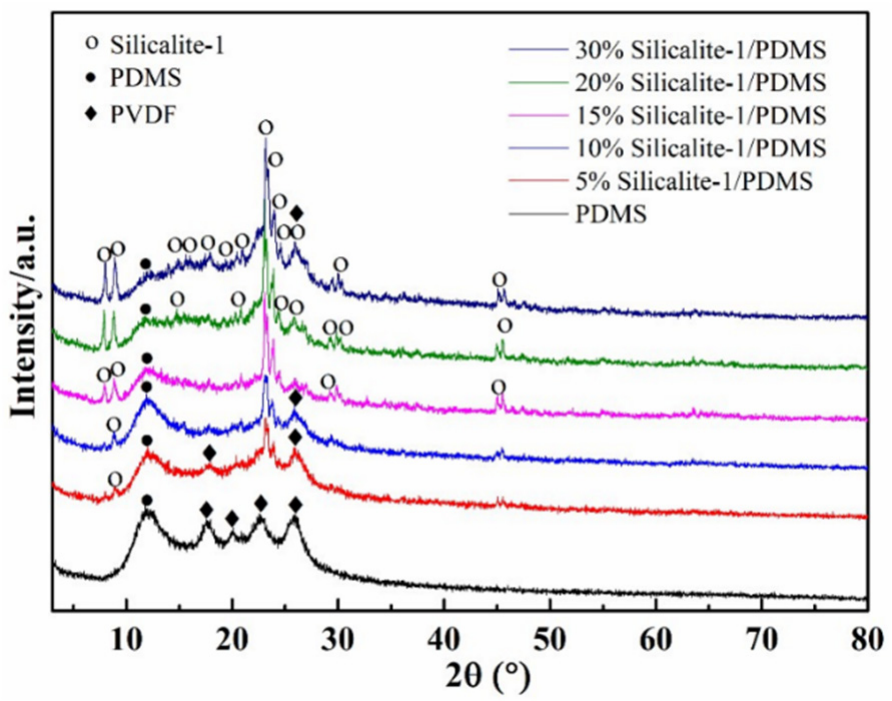
XRD patterns of the silicalite-1/PDMS/PVDF hybrid membranes with different silicalite-1 zeolites contents.

**Figure 8:**
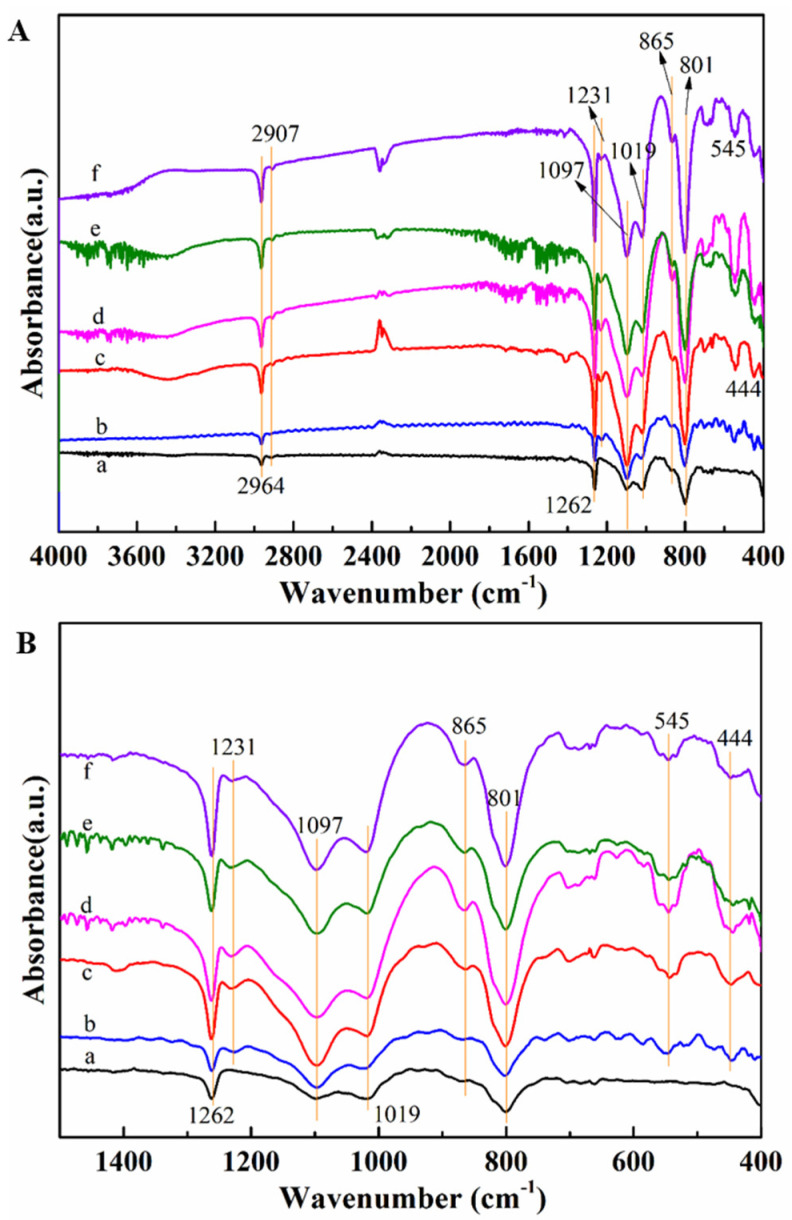
FTIR spectrums of the silicalite-1/PDMS/PVDF hybrid membranes with different silicalite-1 zeolites content: (a) pure PDMS; (b) 5% loading; (c) 10% loading; (d) 15% loading; (e) 20% loading; and (e) 30% loading. (**B**) was the magnified segment of (**A**).

Figure 7 and Figure 8 were the XRD patterns and FTIR spectrums of the silicalite-1/PDMS/PVDF hybrid membranes with different silicalite-1 zeolites contents, respectively. As shown in Figure 7, the characteristic peaks of silicalite-1 zeolites, PDMS and PVDF were all well detected. The broad peak at 2θ = 10~15° corresponding to the diffraction of the PDMS [70]. The peak from 2θ = 15~30° was ascribed to the characteristic peak of the porous PVDF supports, and the peaks at 2θ = 7.9°, 8.9°, 13.2°, 13.8°, 14.7°, 23.0°, 23.3° and 24.0° were the typical diffraction plane of silicalite-1 zeolites. Notably, with the silicalite-1 zeolites contents increasing, the intensity of the silicalite-1 zeolites peaks became stronger, and the characteristic peaks of the PDMS and the PVDF became weaker.For the FTIR spectrums in Figure 8, both the characteristic peaks of PDMS and silicalite-1 zeolites were observed in the corresponding FTIR spectrums. The band at 2964 cm^−1^ was the asymmetrical stretching vibration of CH_3_ [71] and the band at 2907 cm^−1^ represented the stretching vibration of CH_3_ [72]_._ The band appearing at 1262 cm^−1^ was the bending vibration of Si−CH_3_, and the band at 1019 cm^−1^ and 1097 cm^−1^ were the asymmetrical stretching vibration of Si-O-Si, respectively [72]. The sharp band at 801 cm^−1^ was the overlapping peaks about the Si−CH_3_ stretching vibration of PDMS and Si−O−Si symmetric stretching of silicalite-1 zeolites [73]. Moreover, the weak band at 865 cm^−1^ ascribed to the stretching vibration of Si−CH_3_ was also detected [75]. Compared with the FTIR spectrums of pure PDMS membranes in Figure 4b, an additional new peak at 1231 cm^−1^ was observed in the FTIR spectrums of the silicalite-1/PDMS hybrid membranes [77,78]. The band near 545 cm^−1^ was attributed to the double five-ring lattice vibration of the external linkages [79]. The band at 444 cm^−1^ was due to the bending vibration of Si−O−Si internal tetrahedral [80]. The results of the FTIR spectrums indicated that the silicalite-1 zeolites were well enclosed in the PDMS matrix.

### 3.2. Pervaporation Evaluation Experiment

**Figure 9:**
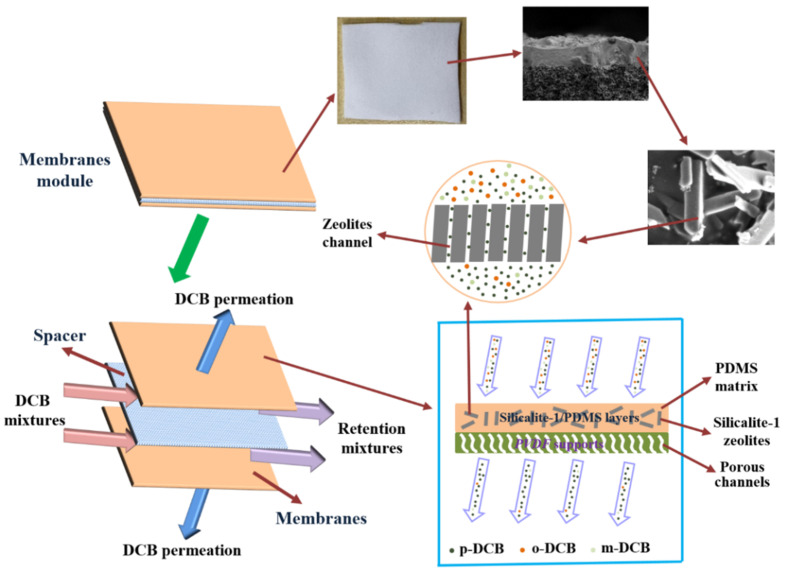
Schematic of the DCB isomers separation mechanism by silicalite-1/PDMS/PVDF hybrid membranes.

**Figure 10:**
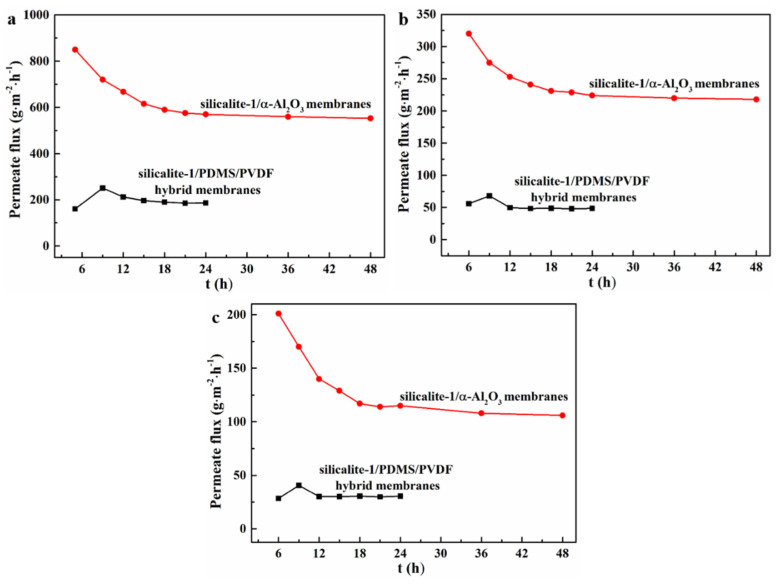
Pervaration flux results of the silicalite-1/α-Al_2_O_3_ membranes [3] and the silicalite-1/PDMS/PVDF hybrid membranes in single-isomer solutions: (**a**) p−DCB; (**b**) o−DCB; and (**c**) m−DCB.

Generally, as a kind of MFI−type zeolites, silicalite-1 zeolites have more uniform and narrower interconnected three−dimensional pore system with the a−directional zig−zag channels (0.51 × 0.57 nm) and the b−directional straight channels (0.54 nm). Theoretically, only the molecular has similar diameter to the channel size of silicalite-1 zeolites can easily enter into the channel, especially p−DCB (kinetic diameter of ~0.58 nm, smaller than o−DCB of ~0.68 and p−DCB of ~0.68) [6]. Thus, silicalite-1 zeolites could be used as active ingredient of silicalite-1/PDMS/PVDF hybrid membranes for separation of individual p−DCB from the mixture of p/o−DCB or p/m−DCB. To our knowledge, crack, large pore, non−zeolite mesopore and micropore, zeolite channel are the five types of pores existing in the active layers of zeolites membranes. As an ideal separation material, adsorption microspore and zeolites channel should be the type of pores existing in the active layers of the silicalite-1/PDMS/PVDF hybrid membranes. Herein, we used 1,3,5−triisopropylbenzene (TIPB, ~0.85 nm) as pervaporation agent to detect the integrity of the silicalite-1/PDMS/PVDF hybrid membranes before evaluation experiment. The integrity test showed extremely small amounts of TIPB were detected in the permeate solutions when using the silicalite-1/PDMS/PVDF hybrid membranes with silicalite-1 content no more than 10%. It indicated the silicalite-1/PDMS/PVDF hybrid membranes with silicalite-1 content no more than 10% exhibited excellent structural integrity. We had reasons to believe that there were no cracks and large pores, and only a minimum number of non−zeolite mesopores or microspores existed in the active silicalite-1/PDMS layers, with silicalite-1 content no more than 10%. However, for the silicalite-1/PDMS/PVDF hybrid membranes with higher silicalite-1 content (>10%), large amounts of TIPB were obviously detected in the permeate solutions. The result demonstrated that the silicalite-1/PDMS/PVDF hybrid membranes of higher silicalite-1 content (>10%) possessed poor structural integrity. Therefore, the silicalite-1/PDMS/PVDF hybrid membranes with higher silicalite-1 content (>10%) were not conducive to the separation of DCB mixtures. Considering the above results, the silicalite-1/PDMS/PVDF hybrid membranes with silicalite-1 content no more than 10% could be used for the pervaporation separation of DCB isomers from their mixtures. In order to evaluate the pervaporation separation property of DCB isomers; we used the silicalite-1/PDMS/PVDF hybrid membranes with 10% zeolites content as the separation material; and then the template synthesized silicalite-1/α−Al_2_O_3_ membranes were used as control group. Figure 9 was the separation mechanism of DCB isomers by silicalite-1/PDMS/PVDF hybrid membranes. The pervaporation flux curves of the silicalite-1/α−Al_2_O_3_ membranes and the silicalite-1/PDMS/PVDF hybrid membranes in single−isomer solutions at 60 °C was shown in Figure 10. Apparently; all the pervaporation flux curves of the silicalite-1/PDMS/PVDF hybrid membranes for p−DCB; o−DCB and m−DCB exhibited similar trend to that of the silicalite-1/α−Al_2_O_3_ membranes; which also included a first rapid growth stage to a maximum flux and then a slow descent stage to a relatively constant value. The above phenomenon could be ascribed to the adsorption−diffusion mechanism; which was similar to that of xylene isomers as the literature reported [32]. The p−DCB; o−DCB and m−DCB were chemically absorbed by the free active site on the surfaces of zeolite internal pores at the first stage; and then blocked the channels of the silicalite-1 zeolites during the pervaporation process. As shown in Figure 10a; the constant permeation fluxes of the silicalite-1/α−Al_2_O_3_ membranes and the silicalite-1/PDMS/PVDF hybrid membranes for the separation of p−DCB were about 553.0 g∙m^−2^∙h^−1^ after 48 h and 185.9 g∙m^−2^∙h^−1^ after 24 h, respectively. Moreover; the constant permeation flux of the silicalite-1/α−Al_2_O_3_ membranes for o−DCB was about 218.2 g∙m^−2^∙h^−1^ after 48 h; and the constant permeate flux of the silicalite-1/PDMS/PVDF hybrid membranes for o−DCB was about 48.5 g∙m^−2^∙h^−1^ after 24 h (Figure 10b). Theoretically, the molecule larger than the pore size of silicalite-1 zeolites cannot pass through the zeolites membranes. However, in our study, the larger molecule o−DCB can also pass through the zeolites membranes. Two reasons might be used to explain this phenomenon. Firstly, the inter-particle mesoporous of silicalite-1 zeolites powders (Figure 2) in the active zeolites layers provided channel for o−DCB transporting. Secondly, the neighboring chloro−-groups distortion of the o−DCB made the molecule smaller, and then could squeeze into the pore channel to permeate [5]. In a previous study, the researchers also demonstrated that both the channel of silicalite-1 zeolites and the structure of the molecular could be distorted when contacting with aromatic medium [81]. Notably, the m−DCB was also detected in the permeate solutions because of the presence of the inter−particle mesoporous of silicalite-1 zeolites powders, as illustrated in Figure 10c. The constant permeation flux of the silicalite-1/α−Al_2_O_3_ membranes for m−DCB was about 106.1 g∙m^−2^∙h^−1^ after 48 h, and the constant permeation flux of the silicalite-1/PDMS/PVDF hybrid membranes for m−DCB was about 30.1 g∙m^−2^∙h^−1^ after 24 h. The constant permeate fluxes of the three isomers were all in the order of p−DCB > o−DCB > m−DCB, and the silicalite-1/α−Al_2_O_3_ membranes had higher constant permeate fluxes than that of the silicalite-1/PDMS/PVDF hybrid membranes.

**Figure 11:**
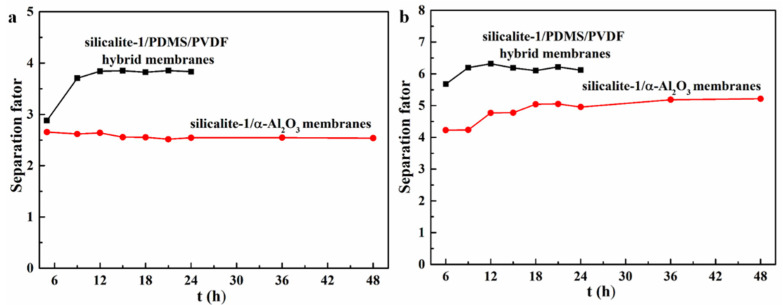
Separation factor results of the silicalite-1/α−Al_2_O_3_ membranes [3] and the silicalite-1/PDMS/PVDF hybrid membranes in single−isomer solutions: (**a**) p/o−DCB; and (**b**) p/m−DCB.

The DCB separation factors of the silicalite-1/α−Al_2_O_3_ membranes and the silicalite-1/PDMS/PVDF hybrid membranes in single-isomer solutions were shown in Figure 11, calculating from the pervaporation results. Obviously, the silicalite-1/PDMS/PVDF hybrid membranes had higher separation factors than that of the silicalite-1/α−Al_2_O_3_ membranes because of the less defects in the active slicalite−1/PDMS layers. For the silicalite-1/PDMS/PVDF hybrid membranes, the separation factors of p/o−DCB and p/m−DCB could reach 3.8 and 6.2, respectively. For the silicalite-1/α−Al_2_O_3_ membranes, the separation factor of p/o−DCB was 2.5 and the separation factor of p/m−DCB was 5.2.

**Figure 12:**
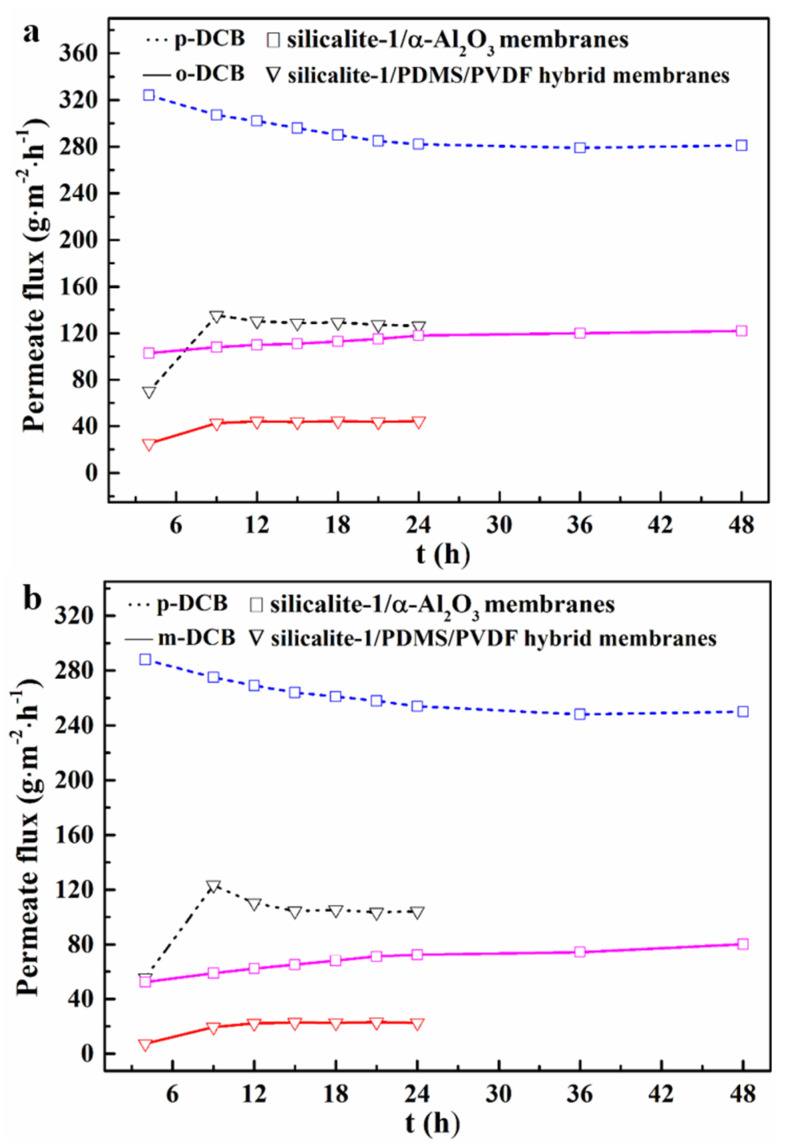
Pervaporation flux results of the silicalite-1/α−Al_2_O_3_ membranes [3] and the silicalite-1/PDMS/PVDF hybrid membranes in binary−isomers solutions (of equal molar content): (**a**) p/o−DCB; (**b**) p/m−DCB.

To further evaluate the separation properties of DCB isomers, pervaporation experiment of equal molar content binary-isomers solutions through the silicalite-1/α−Al_2_O_3_ membranes and the silicalite-1/PDMS/PVDF hybrid membranes was also carried out. Figure 12 was the pervaporation flux curves of the silicalite-1/α−Al_2_O_3_ membranes and the silicalite-1/PDMS/PVDF hybrid membranes in binary−isomers solutions at 60 °C. Both the pervaporation flux curves for p−DCB of the silicalite-1/α−Al_2_O_3_ membranes and the silicalite-1/PDMS/PVDF hybrid membranes in p/o−DCB or p/m−DCB binary−isomers solutions showed a first increasing stage to a maximum flux and then a gradual decreasing stage to a relatively constant value. In contrast to that of p−DCB, the pervaporation flux curves for o−DCB and m−DCB of the above two zeolites membranes in p/o−DCB or p/m−DCB binary−isomers solutions only exhibited a relatively slow increasing stage to a constant flux value. The reason could be attributed to the different affinities between the DCB isomers and the silicalite-1 zeolites, as described in our previously work [5]. After 48 h, the constant permeation flux of the silicalite-1/α−Al_2_O_3_ membranes for p−DCB was about 281.0 g∙m^−2^∙h^−1^ and for o−DCB was about 122.1 g∙m^−2^∙h^−1^ in p/o−DCB binary-isomers solutions (Figure 12a). After 24 h, the constant permeation fluxes of the silicalite-1/PDMS/PVDF hybrid membranes for p−DCB and o−DCB in p/o−DCB binary−isomers solutions were about 126.2 g∙m^−2^∙h^−1^ and 44.5 g∙m^−2^∙h^−1^, respectively. As shown in Figure 12b, the constant permeation fluxes of the silicalite-1/α−Al_2_O_3_ membranes and the silicalite-1/PDMS/PVDF hybrid membranes for p−DCB and m−DCB in p/m−DCB binary-isomers solutions were 250.2 g∙m^−2^∙h^−1^, 80.1 g∙m^−2^∙h^−1^ and 104.3 g∙m^−2^∙h^−1^, 22.7 g∙m^−2^∙h^−1^, respectively. The highest constant permeate flux of p−DCB among the three DCB isomers further indicated that the silicalite-1/PDMS/PVDF hybrid membranes had better ability to separation of p−DCB from the binary−isomers solutions.

In order to compare the pervaporation efficiency of the silicalite-1/α−Al_2_O_3_ membranes (thickness about 5.3 μm) and the silicalite-1/PDMS/PVDF hybrid membranes (average thickness about 17.5 μm), the thickness-normalized pervaporation flux and the thickness-normalized pervaporation separation index were calculated. For silicalite-1/α−Al_2_O_3_ membranes, the thickness−normalized total pervaporation flux was 2.14 μm∙kg∙m^−2^∙h^−1^ and the thickness−normalized pervaporation flux of p−DCB was 1.49 μm∙kg∙m^−2^∙h^−1^ in p/o−DCB binary−isomers solutions. In p/m−DCB binary−isomers solutions, the thickness−normalized total pervaporation flux was 1.75 μm∙kg∙m^−2^∙h^−1^ and the thickness−normalized pervaporation flux of p−DCB was 1.33 μm∙kg∙m^−2^∙h^−1^. For the silicalite-1/PDMS/PVDF hybrid membranes, in p/o−DCB binary−isomers solutions, the thickness−normalized total pervaporation flux and the thickness−normalized pervaporation flux of p−DCB were 2.99 μm∙kg∙m^−2^∙h^−1^ and 2.21 μm∙kg∙m^−2^∙h^−1^, respectively. In p/m−DCB binary−isomers solutions, the thickness−normalized total pervaporation flux was 2.22 μm∙kg∙m^−2^∙h^−1^ and the thickness−normalized pervaporation flux of p−DCB was 1.83 μm∙kg∙m^−2^∙h^−1^. The detailed results of thickness−normalized pervaporation flux were shown in Table 2. Additionally, for silicalite-1/α-Al_2_O_3_ membranes, the thickness−normalized pervaporation separation index of p−DCB in p/o−DCB binary−isomers solutions and that of p−DCB in p/m−DCB binary-isomers solutions were 1.94 μm∙kg∙m^−2^∙h^−1^ and 2.78 μm∙kg∙m^−2^∙h^−1^, respectively. For the sillicate−1/PDMS/PVDF hybrid membranes, the thickness−ormalized pervaporation separation index of p−DCB in p/o−DCB binary−isomers solutions was 4.20 μm∙kg∙m^−2^∙h^−1^ and that of p−DCB in p/m−DCB binary-isomer solutions was 6.57 μm∙kg∙m^−2^∙h^−1^. The values of thickness−normalized pervaporation separation index of p−DCB in p/o−DCB binary-isomers solutions and in p/m−DCB binary-isomers solutions for the sillicate−1/PDMS/PVDF hybrid membranes were, respectively, 2.2 and 2.4 times comparing to the silicalite-1/α−Al_2_O_3_ membranes.

**Figure 13:**
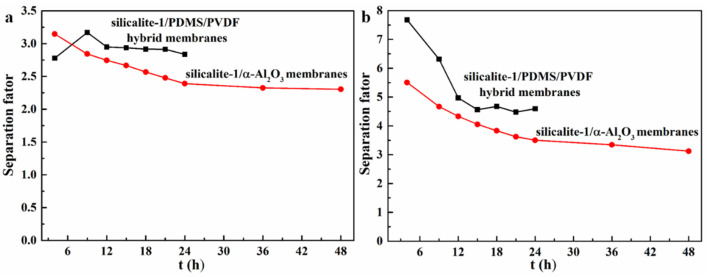
Separation factor results of the silicalite-1/α−Al_2_O_3_ membranes [3] and the silicalite-1/PDMS/PVDF hybrid membranes in binary−isomer solutions (of equal molar content): (**a**) p/o−DCB; (**b**) p/m−DCB.

The DCB isomer separation factors of the silicalite-1/α−Al_2_O_3_ membranes and the silicalite-1/PDMS/PVDF hybrid membranes in binary−isomers solutions were shown in Figure 13. The silicalite-1/PDMS/PVDF hybrid membranes had higher separation factors than that of the silicalite-1/α−Al_2_O_3_ membranes. The separation factors of the silicalite-1/PDMS/PVDF hybrid membranes for p/o−DCB and p/m−DCB were 2.9 and 4.6, respectively. The separation factor of the silicalite-1/α−Al_2_O_3_ membranes for p/o−DCB was 2.3 and for p/m−DCB was 3.1. Considering the above results, we believed that the silicalite-1/PDMS/PVDF hybrid membranes were more suitable for the pervaporation separation of DCB isomers. However, to realize future industrial application, the lifetime of the silicalite-1/PDMS/PVDF hybrid membranes and large−scale membrane preparation technology should be studied.

## 4. Conclusions

The dense and continuous silicalite-1/PDMS hybrid membranes on the porous PVDF supports without longitudinal cracks and any other defects was successfully prepared by scraping method. The SEM images revealed the active silicalite-1/PDMS layers were tightly and properly adhered on the surfaces of the porous PVDF supports. With the zeolites loading increasing, the surfaces of silicalite-1/PDMS/PVDF hybrid membranes became rougher, and more silicalite-1 zeolites aggregated to form pile pores. The optimum silicalite-1 zeolites content was 10%, and the thickness of the active silicalite-1/PDMS layers was 10–15 μm. The pervaporation experiment demonstrated that both the silicalite-1/α−Al_2_O_3_ membranes and the silicalite-1/PDMS/PVDF hybrid membranes had the ability to separate DCB from DCB isomers mixtures. The permeate fluxes of the DCB isomers increased in the order of m−DCB < o−DCB < p−DCB both in single-isomer and binary−isomers solutions for the silicalite-1/α−Al_2_O_3_ membranes and the silicalite-1/PDMS/PVDF hybrid membranes. Comparatively, the silicalite-1/PDMS/PVDF hybrid membranes exhibited higher selectivity and lower pervaporation fluxes for DCB isomers than that of the silicalite-1/α−Al_2_O_3_ membranes. The separation factors of the silicalite-1/PDMS/PVDF hybrid membranes for p/o−DCB and p/m−DCB were 2.9 and 4.6, respectively. The total permeation fluxes of the silicalite-1/PDMS/PVDF hybrid membranes in p/o−DCB and p/m−DCB binary−isomers solutions were 170.7 g/(m^2^∙h) and 127.0 g/(m^2^∙h), respectively. The thickness-normalized pervaporation separation index ratio for the sillicate−1/PDMS/PVDF hybrid membranes comparing to the silicalite-1/α−Al_2_O_3_ membranes in p/o−DCB binary_−_isomers solutions was 2.2 and in p/m−DCB binary−isomers solutions was 2.4. The results suggested that the silicalite-1/PDMS/PVDF hybrid membranes had great potential for future industrial application in pervaporation separation of DCB from their mixtures.

## Figures and Tables

**Table 1:** Results from N_2_ sorption measurements for the silicalite-1 zeolites powders.

Zeolites Sample	S_BET_ (m^2^/g)	S_mic_ (m^2^/g)	S_ext_ (m^2^/g)	V_mic_ (cm^3^/g)	V_meso_ (cm^3^/g)	D_aver_ (nm)	V_total_ (cm^3^/g)
Silicalite-1	356.1	296.4	59.7	0.171	0.038	2.31	0.209
ZSM−5 (SiO_2_/Al_2_O_3_ = 300)	356.0	186.1	169.9	0.090	0.145	2.68	0.235

S_BET_: BET specific surface area (m^2^/g); S_mic_: micropore specific surface area (m^2^/g); S_ext_: external specific surface area (m^2^/g); V_mic_: micropore volume (cm^3^/g); V_meso_: mesopore volume (cm^3^/g); V_total_: total volume (cm^3^/g); D_aver_: average diameter (nm).

**Table 2:** Thickness−normalized pervaporation fluxes for silicalite-1/α−Al_2_O_3_ membranes and the sillicate−1/PDMS/PVDF hybrid membranes.

	p/o−DCB Mixtures			p/m−DCB Mixtures		
	*J_N,p/o−DCB_*	*J_N,p−DCB_*	*J_N,o−DCB_*	*J_N,p/m−DCB_*	*J_N,p−DCB_*	*J_N,m−DCB_*
Silicalite-1/α−Al_2_O_3_ membranes(μm∙kg∙m^−2^∙h^−1^)	2.14	1.49	0.65	1.75	1.33	0.42
Sillicate−1/PDMS/PVDF hybrid membranes (μm∙kg∙m^−2^∙h^−1^)	2.99	2.21	0.78	2.22	1.83	0.40

## Data Availability

The data presented in this study are available on request from the corresponding author upon reasonable request.
